# Expression of *Drosophila virilis* Retroelements and Role of Small RNAs in Their Intrastrain Transposition

**DOI:** 10.1371/journal.pone.0021883

**Published:** 2011-07-11

**Authors:** Nikolay V. Rozhkov, Elena S. Zelentsova, Natalia G. Shostak, Michael B. Evgen'ev

**Affiliations:** 1 Institute of Molecular Biology, Moscow, Russia; 2 Institute of Cell Biophysics, Puschino, Russia; Virginia Tech Virginia, United States of America

## Abstract

Transposition of two retroelements (*Ulysses* and *Penelope*) mobilized in the course of hybrid dysgenesis in *Drosophila virilis* has been investigated by *in situ* hybridization on polytene chromosomes in two *D. virilis* strains of different cytotypes routinely used to get dysgenic progeny. The analysis has been repeatedly performed over the last two decades, and has revealed transpositions of *Penelope* in one of the strains, while, in the other strain, the LTR-containing element *Ulysses* was found to be transpositionally active. The *gypsy* retroelement, which has been previously shown to be transpositionally inactive in *D. virilis* strains, was also included in the analysis. Whole mount *is situ* hybridization with the ovaries revealed different subcellular distribution of the transposable elements transcripts in the strains studied. *Ulysses* transpositions occur only in the strain where antisense piRNAs homologous to this TE are virtually absent and the ping-pong amplification loop apparently does not take place. On the other hand small RNAs homologous to *Penelope* found in the other strain, belong predominantly to the siRNA category (21nt), and consist of sense and antisense species observed in approximately equal proportion. The number of *Penelope* copies in the latter strain has significantly increased during the last decades, probably because *Penelope*-derived siRNAs are not maternally inherited, while the low level of *Penelope-*piRNAs, which are faithfully transmitted from mother to the embryo, is not sufficient to silence this element completely. Therefore, we speculate that intrastrain transposition of the three retroelements studied is controlled predominantly at the post-transcriptional level.

## Introduction

Transposable elements (TEs) are repetitive sequences capable of moving in genomes under certain conditions, and they are widely observed in practically all organisms studied so far. The diversity of TEs and the degree to which they burden eukaryotic genomes are highly variable. In mammals, including humans, mobile genetic elements constitute up to 50% of the genome [1], while only 15–20% of the comparatively small Drosophila genome is composed of TEs [2]. Different classes of transposons, such as LTR-containing retroelements, LINEs and DNA transposons, are also represented to different degrees in the genomes of various organisms. Host organisms employ multiple strategies to silence TEs and viruses to prevent them from amplifying in the genome, because the vast majority of parasite insertions are likely to be deleterious and impose a fitness cost on the rest of the genome [3], [4]. Recent data accumulated from *Ceanorharbditis elegans* and Drosophila, strongly suggest that RNA interference represents one of the most efficient host processes for silencing transcription and uncontrolled movement of parasite DNA [5], [6], [7]. Even though eukaryotic genomes have developed multiple systems for silencing TEs, certain families of TEs sometimes go out of control and are able to amplify and jump throughout the chromosomes [8]. The hybrid dysgenesis (HD) syndrome, described in *Drosophila melanogaster* and *Drosophila virilis,* represents such a case, where multiple transpositions of TEs lead to harmful consequences [9], [10].

In *D. melanogaster* the HD syndrome is usually observed in the progeny of interstrain crosses when the female parent does not carry active copies of a certain TE (*P*, *I* or *hobo*), while the male parent carries multiple copies of a given element. Briefly, in *D. melanogaster* the dysgenic traits in the F1 progeny from a dysgenic cross usually include high levels of sterility, gonadal atrophy, occurrence of multiple visible and chromosomal mutations, and other genetic abnormalities. Although in *D. virilis* we observed virtually the same abnormalities, HD syndrome in this species is unusual in the fact that several transposable elements belonging not only to different families but also to different classes of TE are mobilized by the dysgenic crosses [10], [11], [12]. In our earlier studies, we showed that in *D. virilis,* similar to *D. melanogaster,* there are strains of three cytotypes, namely, neutral, M-like and P-like strains, depending upon their roles in HD [11]. In *D. melanogaster* strains of M-cytotype do not contain functional *P*-elements and produce partially sterile progeny when crossed with males from P-strains carrying multiples copies of full-size *P*-elements while neutral strains do not produce significant proportion of sterile progeny when crossed either with M-like or P-like strains [9]. In *D. virilis* strains named by analogy with *D. melanogaster* “M-like strains”, including the wild-type strain 9 used in the present study, usually contain only heterochromatic, highly diverged copies of *Penelope* retroelements. Furthermore, such diverged copies of *Penelope* are located in such strains mainly in the pericentromeric heterochromatin [13]. These strains produce high levels of gonadal sterility and other manifestations of HD when crossed with males of strain 160, which represents the only strong P-like strain described in *D. virilis* so far and contains multiple copies of *Penelope* probably playing an important role in HD [10]. *In situ* hybridization on polytene chromosomes and Southern blot analysis revealed mobilization of several unrelated TEs in the progeny of dysgenic crosses. These elements include *Helena*, *Paris*, *Tv1*, *Telemac*, *Ulysses* and *Penelope* [12], [14]. Among these, *Ulysses* which represents a typical retroelement with LTRs of 2 kb in size and two ORFs, was the first element described in *D. virilis* and subsequently found in several visible mutations, including *white*, obtained in the progeny of dysgenic crosses [10], [14]. Furthermore, this element was found at the breakpoints of inversions detected in the progeny of dysgenic crosses and, hence, it was implicated in the formation of aberrations never before found in *D. virilis* [15]. In contrast to *Ulysses*, another well studied LTR-containing retroelement g*ypsy,* previously described in *D. virilis* (g*ypsyDv*) [16], was never found in mutations in the progeny of dysgenic crosses [12].

It has been shown by different methods that multiple active copies of *Penelope* are present in strain 160, while strain 9 does not carry full-size *Penelope* copies in the euchromatic chromosome arms [10], [17]. Highly diverged and apparently ancient copies of *Penelope,* termed “Omega” (Ω), located mostly in the heterochromatic chromocenter, were, however, detected and investigated in both strains studied [13]. *In situ* hybridization with polytene chromosomes and Southern blotting analysis showed that contrary to *Penelope*, full-size *Ulysses* copies are found in all *D. virilis* strains studied so far, with an average of 10–15 copies per strain [18].

There is molecular and genetic evidence suggesting that the TE “*Penelope*” plays an important role in *D. virilis* HD [10], [17]. The *Penelope* retroelement does not belong to one of the previously well studied classes of TE, but rather represents its own superfamily characterized by the presence of a reverse transcriptase (more closely related to telomerases than the those of other retrotransposons) and a very unusual endonuclease containing the GIY-YIG domain [13]. *Penelope*-like elements (“PLEs”) have been described in recent years in various animals from rotifers to fish and reptiles [19], [20], [21].

In our previous studies, the injection of *Penelope*-containing constructs into the embryos of a *D. virilis* strain 9 lacking active *Penelope* resulted in multiple mutations in the progeny. It was shown that almost half of all visible mutations isolated in these experiments were due to insertions of *Ulysses* [10], which, contrary to *Penelope*, has nearly symmetrical distribution in the parental strains [18].

Recently, we have monitored the biogenesis of small RNAs homologous to various *D. virilis* transposons and measured the transmission levels of corresponding siRNAs and piRNAs in various inter-strain crosses. Using P-like strain 160 and a few neutral *D. virilis* strains that contain multiple full-size and potentially functional *Penelope* copies, however, we detected no obvious correlation between dysgenic traits and maternally deposited *Penelope*-derived piRNA levels [22]. Therefore, we sought to expand these studies in order to reveal correlations between the levels of naturally occurring transposition in *D. virilis* laboratory strains and RNA production and/or the biogenesis of the TE-derived small RNAs in question.

Herein, we demonstrate asymmetric transposition of *Penelope* and *Ulysses* in the laboratory strains of *D. virilis* without performing dysgenic crosses. By RNA whole-mount *in situ* hybridization a different subcellular strain specific localization of the TEs transcripts was revealed. Furthermore, we show that processing of *Penelope* and *Ulysses* transcripts lead to the formation of different classes of small RNAs that may be implicated in transposition control of these TEs. For comparison, we have also investigated expression of *gypsyDv*, which is based upon previous studies lost transposition activity in *D. virilis* and is not mobilized by dysgenic crosses in this species [12].

## Results

### Analysis of transpositions of *Penelope* and *Ulysses* in two *D. virilis* strains by *in situ* hybridization on polytene chromosomes

In the course of investigations performed over the past 20 years we detected asymmetric transpositions of *Penelope* and *Ulysses* in *D. virilis* strain 160 and strain 9. Using *in situ* hybridization with salivary gland polytene chromosomes, we failed to detect any transpositions of *Ulysses* in strain 160, which preserved stable pattern of the transposon distribution in the chromosomes. On the other hand, the number of *Penelope* copies in this strain increased from 37 to 53 since 1991. We detected 27 new sites of *Penelope* hybridization and the disappearance of 11 previously observed sites in the chromosomes of the strain 160. Interestingly, nearly half of the new sites–12–were found in the chromosome 2 (Table 1). It is noteworthy that we failed to detect *Penelope* hybridization to chromosome 6 (microchromosome), not only in strain 160, but also in all other *D. virilis* strains studied in our laboratory [18].

**Table 1:** Copy number of *Penelope*, *Ulysses* and *gypsyDv* in polytene chromosomes of *D. virilis* strains 9 and 160.

		Time of analysis			
		1991–1992		2008	
Chromosome	Transposon	strain 9	strain 160	strain 9	strain 160
**X**	*Penelope*	-	1D, 8D, 9D, 10B, 11A, 18C	-	6C, 8D, 9C, 10B, 11A, 12C
	*gypsyDv*	11A, 19D	11A, 18D, 19D	11A, 19D	11A, 18D, 19D
	*Ulysses*	17D/18A, 19D	18B, 19C, 19D	2C, 9A, 17D/18A, 19D	18B, 19C, 19D
**2**	*Penelope*	-	20E, 20 F/G, 22D, 23F, 28F	-	20E/F, 22D, 23B, 23D, 23F, 25D, 24B, 26F, 27D/E, 27E, 27G, 29B (2 sites), 29C, 29H
	*gypsyDv*	23CD	-	23C	-
	*Ulysses*	21A, 24 B/C, 25F/G, 26C, 29F	23H, 29D	20D, 21A, 22E, 24B, 25F/G, 26C	23H, 29D
**3**	*Penelope*	-	30A, 32F, 34F, 35B, 37C, 38A, 38E/F, 38F, 39A/B, 39E, 39F	-	30A, 32A, 32C, 32F, 33B/C, 33E, 34F, 35B, 37C, 38E/F, 39A/B, 39E, 39F
	*gypsyDv*	39F	39F	39F	39F
	*Ulysses*	33C, 34A, 37D/E	32A/B, 35E	33C, 34A	32A/B, 35E
**4**	*Penelope*	-	40B, 40E, 42C, 45B, 45F, 46B, 46E/F, 47A, 49F*	-	40B, 40E, 40F, 42C, 44C, 45B, 45D, 45F, 46B, 46E, 47A, 49F*
	*gypsyDv*	49F*	46B, 49F*	49F*	46B, 49F*
	*Ulysses*	42C, 49F*	49F*	40B, 42C, 49F*	49F*
**5**	*Penelope*	-	51A, 52E, 55F, 56F, 58F, 59C	-	50D/E, 51A, 51E, 57B, 57D, 58F, 59F
	*gypsyDv*	-	-	-	-
	*Ulysses*	51C, 52D, 52E, 53B, 55D, 58F	53C, 53F, 54C, 55F, 59F	51C, 52D, 52E, 53B	53C, 53F, 54C, 55F, 59F
**6**	*Penelope*	-	-	-	-
	*gypsyDv*	60CD	60CD	60CD	60CD
	*Ulysses*	60C	60A, 60B/C	60A, 60C	60A, 60B/C

Copy number was determined by *in situ* hybridization analysis within the last two decades (1991–2008). When performing *in situ* hybridization analysis in 2008, we excluded a few *Ulysses* sites that were polymorphic in 1991 (did not contain *Ulysses* in 100% of larvae). Asterisks indicate site 49F where all three TEs were found.

While we did not find new sites for *Ulysses* in strain 160, we did reveal active transposition of this TE in M-like strain 9. It is noteworthy that all the chromosomes of strain 9 were involved in the transposition process by *Ulysses* (Table 1). It is necessary to note that even though transpositions of retroelements do not occur by a “cut and paste” mechanism, in strain 9 we detected six new sites of insertion in parallel with the disappearance of four “old” sites detected in 1991. Such a phenomenon was described in *D. melanogaster*, when certain copies of the retroelement *gypsy* or *I*-element disappeared without a trace from a few cytological locations [23], [24].

Characteristically, the presumably inactive *gypsyDv* taken for comparison exhibited practically identical preferentially heterochromatic distribution in the chromosomes of the *D. virilis* strains studied, which was preserved without any change during the whole period of observation (Table 1). It is noteworthy that a vast majority of the same, probably heterochromatic, cytological sites contain *gypsyDv* in all other laboratory and geographic *D. virilis* strains studied so far (data not shown), which implies that this TE has probably lost its transposition ability in this species. It is noteworthy that both *Ulysses* and *gypsyDv* are often found in nearcentromeric sites (i.e. 19D, 29F, 39F, 49F and 59F) while *Penelope* with one exception are not found in these presumably heterochromatic regions (Table 1).

Since we detected different transposition behavior of *Penelope* and *Ulysses* depending upon the strain, it was of significant interest to monitor the transcription of various TEs, including these retroelements, in the strains compared.

### Transcription analyses of various TEs and transcripts subcellular localization

**Figure 1:**
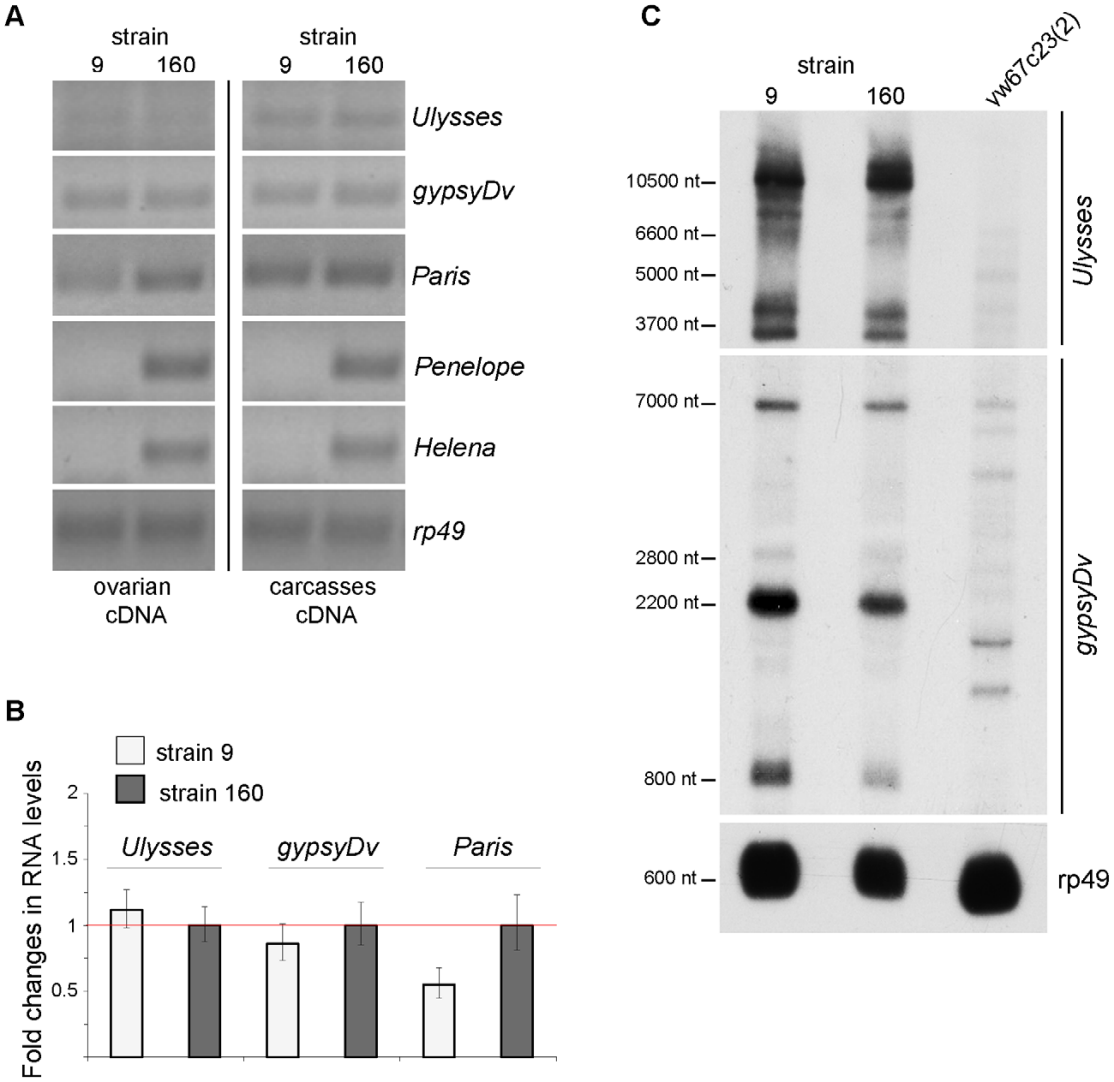
Transcription levels of selected *D. virilis* TEs. (A) semiquantitative RT-PCR data for ovaries and carcasses; (B) Quantitative RT-PCR analysis of TE transcription levels in ovaries. Since RT-PCR failed to reveal any transcription of *Penelope* and *Helena* in strain 9, we do not include the results of comparative analysis of these TEs by qRT-PCR in the panel; (C) Northern blot detection of *Ulysses* and *gypsyDv* sense transcripts in strains 9 and 160. Poly-A RNAs isolated from strain 9, strain 160 and *D. melanogaster* yw^67c23^ strain ovaries were used. The size of marker RNA is given in nt at the right. The filter was rehybridized with a fragment of constitutively expressed *D. melanogaster rp49* to monitor the level of loaded RNA.

Previously it was shown by Northern analysis that *Penelope* transcription is significantly induced in the ovaries of dysgenic hybrids between females of strain 9 and males of strain 160 [10]. However, transcription of other TEs has not been analyzed either in parental strains 9 and 160 or in their hybrids. Therefore, we explored a transcription of several transposons by a set of complementary methods. In order to detect a presence of transcripts, semiquantitative RT-PCR was performed on cDNA from both strains with specific primers to the transposons. It was also of interest to investigate the transposon transcription separately in female gonads and carcasses. RT-PCR products were detected for all elements studied both in ovaries and in the carcasses, but the presence of some transcripts was shown to be strain specific (Figure 1A). Thus, *Penelope* and *Helena* transcripts were amplified only in strain 160, what is not unexpected, because functional copies of these particular TEs are not present in strain 9 [12]. On the other hand, *Ulysses* and *gypsyDv* are transcribed in ovaries and in the carcasses of both strains 9 and 160 (Figure 1A).

Surprisingly, quantitative RT-PCR (qRT-PCR) experiments revealed a comparatively low but significant level of *Paris* transcription in strain 9, while full-size copies of this TE were previously reported either to be lacking or represented by only one euchromatic copy in this strain [12], [17]. The transcripts detected may either emerge from this single copy or result from read through transcription of *Paris* heterochromatic diverged sequences. qRT-PCR experiments demonstrated that the transcription levels of *Ulysses* and *gypsyDv* in strain 9 are similar to those in strain 160 (Figure 1B).

Northern blot analysis corroborates the qRT-PCR data, and demonstrates approximately the same level of *Ulysses* and *gypsyDv* transcription in the ovaries of both strains. Moreover RNAs homologous to these TEs are represented in Northern blots by identical bands (presumably splicing forms) in both strains (Figure 1C).

It is noteworthy that *Ulysses* probe did not reveal any significant hybridization with *D. melanogaster* RNA because the representatives of *Ulysses* family are absent in the genome of this species. *D. virilis gypsy* probe hybridized with *D. melanogaster* RNA and revealed full-size transcript (7 kb) and several additional bands probably resulted from splicing (Figure 1C).

**Figure 2:**
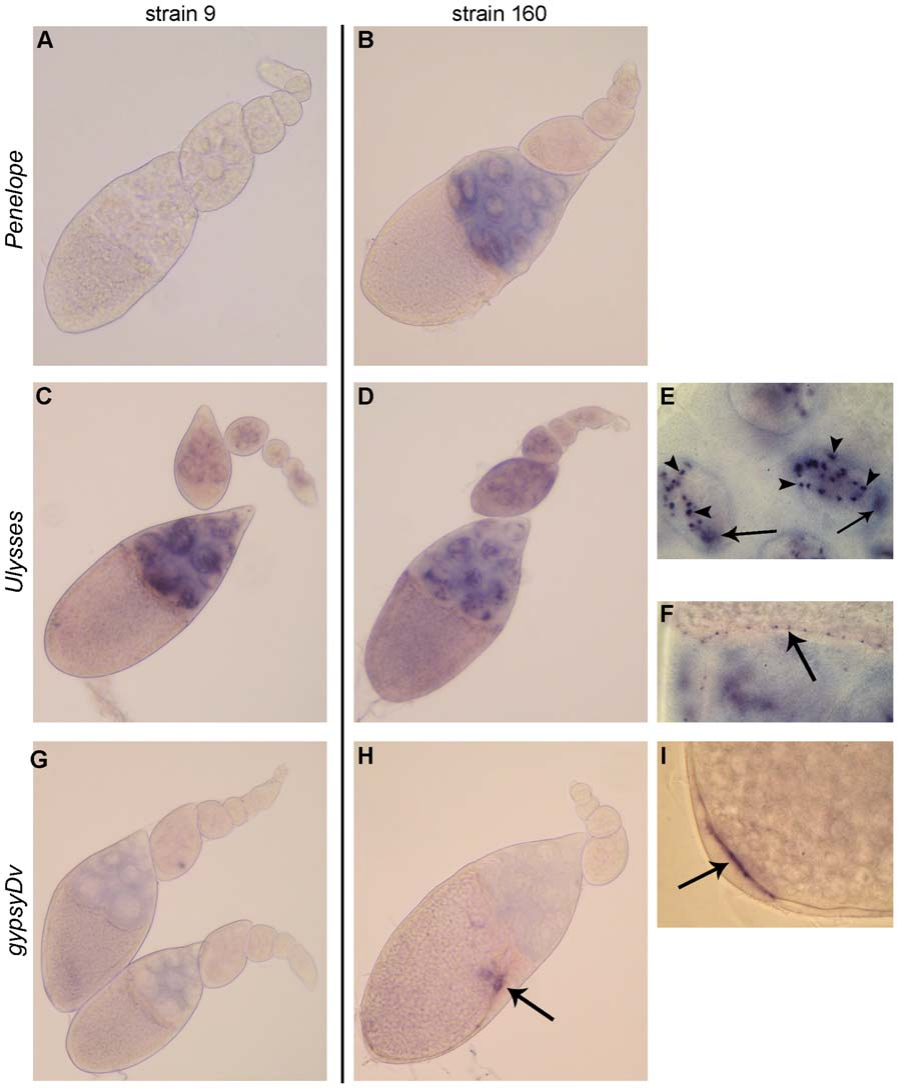
Whole-mount *in situ* RNA detection of sense transcripts of *Penelope*, *Ulysses* and *gypsyDv* in the ovaries of *D. virilis* strains 9 and 160. (A) and (B) hybridization with *Penelope*-specific probe. No hybridization is seen in strain 9 (A), while in strain 160 (B) strong hybridization in the nurse cells cytoplasm is evident at stage 10. *Ulysses*-specific probe strongly hybridized with nurse cells nuclei in both strains at stages 2–10 (C, D, E). Arrows in E indicate putative RNA processing sites (foci), arrow-heads indicate putative nascent transcripts. Heavier label accumulation is usually observed in the cytoplasm of nurse cells of strain 9 (C). Reproducible hybridization of *Ulysses* probe with the centripetal (see arrow in F) and stretched follicle cells is a characteristic feature of strain 9 and 160 ovaries at stage 10. RNA in situ hybridization with *gypsyDv*-specific probe reveals hardly detectable labeling in the nurse cells cytoplasm in the ovaries of both strains studied (G–I). Significant hybridization of *gypsyDv* probe with follicle cells, which form appendages (H) and with follicle cells at the posterior end of ovarian chamber (I) represent the landmarks of strain 160.

At the next stage, in order to monitor subcellular localization of TE transcripts in the ovaries, we performed RNA *in situ* hybridizations with *Penelope, Ulysses* and *gypsyDv* sense and antisense probes. Localization of *Penelope* sense transcripts is shown in Figure 2A, B. It is evident that strong hybridization of *Penelope* in strain 160 is restricted to the cytoplasm of nurse cells, and, to a lesser extent, to the nuclei of nurse cells, while the ovaries of strain 9 do not contain any label, as expected. We observed only very weak hybridization with a probe revealing antisense *Penelope* transcript in strain 160 (data not shown). In contrast to *Penelope*, a probe revealing sense transcript of *Ulysses* detected multiple signals in the nuclei of nurse cells in both strains in the form of discrete putative nascent transcripts as well as single strong signals (one per nucleus) probably representing RNA processing sites (foci) (Figure 2E). This hybridization pattern resembles *I*-element localization in *D. melanogaster* ovaries [25]. Interestingly, in strain 9 the labeling seems to be more pronounced in the cytoplasm of nurse cells in comparison with strain 160 (Figure 2C, D). On the other hand, in approximately 90% of strain 160 ovarioles, quite distinct large foci close to the nuclear membrane are seen (Figure 2E). These structures, where in *D. melanogaster* accumulation of *I*-element and other TEs transcripts takes place, probably represent the sites of processing of various TEs RNAs leading to their retention in the nuclei [25]. Characteristically, the foci are never seen in the ovarioles of strain 9, which correlates with the active transposition of *Ulysses* in strain 9. A significant signal is also observed in the cytoplasm of centripetal and squamous follicle cells in the ovaries of both strains (Figure 2F). Whole mount detection of sense *gypsyDv* transcripts revealed rather weak hybridization in the cytoplasm of nurse cells in both strains (Figure 2G), while the somatic follicle cells were practically free of label with a few specific exceptions (Fig. 2H, I). The pattern observed in *D. virilis* is strikingly different from subcellular localization of *gypsy* transcripts in ovaries of *D. melanogaster* permissive strains [26].

Overall, the analysis of the transcription of various TEs, including *Penelope, Ulysses* and *gypsyDv* performed by different complementary techniques in the strains compared, revealed characteristic differences in the TE's RNA levels and transcript localization in the cells of the ovaries. In order to further investigate the fate of TEs transcripts, we decided to perform detailed analysis of small RNAs homologous to the retroelements studied.

### *Penelope* and *Ulysses* produce strikingly different sets of of transposon-homologous small RNAs in gonads of strains 9 and 160

Since in contrast to *Penelope* we failed to reveal a clear-cut correlation between the expression levels of *Ulysses* and *gypsyDv* and their transposition behavior in both strains we investigated the biogenesis of small RNAs homologous to these TEs.

**Figure 3:**
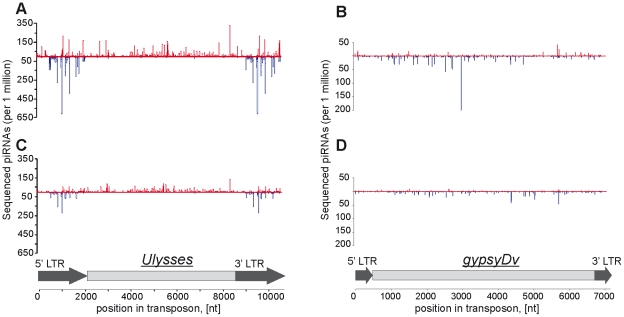
The pattern of piRNAs distribution along transposons in testes. Distribution of *Ulysses*-piRNAs in testes of strain 9 (A) and strain 160 (C). The distribution of piRNAs homologous to *gypsyDv* in testes of strain 9 (B) and strain 160 (D). Sense small RNAs are indicated in red, antisense – in blue.

In order to evaluate the possible role of small RNAs, such as pi- and siRNAs, in controlling the detected transpositions of *Penelope* and *Ulysses*, we explored small RNA libraries obtained from the ovaries and testes of strains 9 and 160 [22]. The analysis of small RNA populations homologous to *Penelope* and *Ulysses* in these strains revealed drastic differences in the processing of their transcripts. While *Ulysses*-derived small RNAs in both strains are represented by predominantly piRNAs 23–29 nt in length (92% of total reads), *Penelope*-homologous small RNAs are virtually absent in strain 9, and in strain 160 they mostly belong to the siRNA species 21 nt in length (61% of total reads) [22]. It is well known that the phenomenon of RNA interference is based on homology-dependent gene silencing, and since we detected full-length sense transcripts of *Ulysses* (Figure 1C), it was logical to expect that *Ulysses*-derived small RNAs will have an antisense orientation. However, contrary to the expectation, up to 99% of the ovarian piRNAs homologous to *Ulysses* have sense orientation and apparently arise from processing transcripts originating from active euchromatic copies of the element [22]. Surprisingly, a high level of *Ulysses*-derived antisense piRNAs was found in the small RNAs libraries obtained from the testes of both strains. Moreover, the piRNAs are predominantly homologous to the sequences of the TE's huge LTRs (Figure 3A, C). *gypsyDv*-piRNAs are represented predominantly by antisense population in both the ovaries and the testes. In this case the LTRs of this TE are not enriched in piRNAs, while general pattern of piRNA localization along the body of this TE is very similar in ovaries and testes but is different depending on a strain (Figure 3B, D).

**Figure 4:**
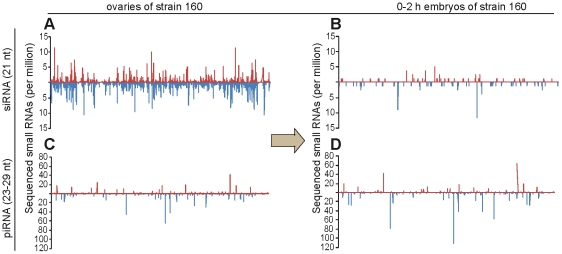
Maternal deposition and distribution levels of *Penelope*-derived small RNAs. siRNAs at (A, B) and piRNAs at (C, D) in strain 160 and its 0–2 h embryos. Sense small RNAs are indicated in red, antisense–in blue.

There is evidence suggesting an important role of maternally inherited small RNAs in repression of *I*- and *P*-elements or *Penelope* in HD syndrome [27], [28]. However, in the latter case it was not shown which class of small RNAs is responsible for the effect. Here, we monitored separately the maternal deposition of siRNA and piRNA of *Penelope* in the strains compared. The experiments showed that although *Penelope* siRNAs are present at high level in the ovaries of both strains, this class of small RNA is practically absent in 0–2 hour embryos (Figure 4A, B). On the contrary, *Penelope*-derived piRNAs as expected are effectively transmitted to the progeny (Figure 4C, D). It is necessary to mention that *Penelope* transcripts are detected in ovaries of strain 160 and maternally inherited by the early embryos (0–2 hours) [29].

**Figure 5:**
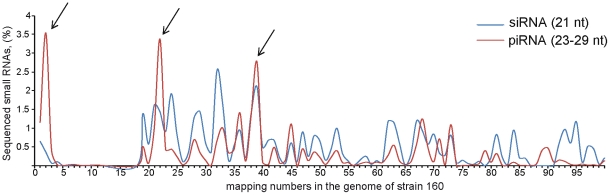
Frequency distribution of genomic mappings of *Penelope*–homologous si- and piRNAs. Arrows indicate a proportion of *Penelope*-derived piRNA sequences mapping 2, 22 and 39 times in *D. virilis* genome.

It was of significant interest to compare the data accumulated in the course of *in situ* hybridization studies on polytene chromosomes with the results of mapping sequenced small RNAs homologous to *Penelope* and *Ulysses*. The comparative analysis has shown that large part of *Ulysses*-derived piRNAs map to the *D. virilis* genome 17–21 times [22]. Similarly, a significant proportion of *Penelope*-derived piRNA sequences map 2, 22 and 39 times while we did not detect sequences found in the genome 3-18 times (Figure 5). Probably, rearranged or full-length transcribed *Penelope* elements as well as ancient diverged copies (Omega) located in heterochromatic clusters may serve as the source of the multiple piRNAs homologous to the element in strain 160.

## Discussion

In our experiments, we showed that *Penelope* and *Ulysses* are able to asymmetrically transpose in *D. virilis* parental strains even without performing dysgenic crosses that drastically increase the frequency of unrelated TE transpositions in this species. It is necessary to mention that transpositions of various TEs were detected in laboratory strains of *D. melanogaster* and sometimes the intrastrain mobility of certain TEs correlates with their expression level [24], [30].

In our study, we observed similar levels of *Ulysses* transcription in both *D. virilis* strains while this TE is transpositionally active only in strain 9. Furthermore, although *gypsyDv* full-size transcripts are present in both strains, this element is amazingly stable in terms of transposition.

Whole-mount *in situ* hybridization experiments demonstrated different subcellular localization of TEs transcripts, which in general correlates with their transposition behavior in the strains studied. Contrary to *D. melanogaster* permissive strains, *gypsyDv* is weakly expressed only in the nurse cell cytoplasm and specific groups of follicular cells in both strains what correlates with its stability in the genome of *D. virilis*. Abundant *Penelope* transcripts were observed in the cytoplasm of nurse cells of strain 160, where this TE is probably transpositionally active. Furthermore, while *Ulysses* expression in the form of strong nascent transcripts has been detected in the nurse cells of both strains, only in strain 160 well developed foci (presumptive sites of *Ulysses* RNA processing) were seen correlating with high stability of *Ulysses* localization in this strain.

The transposition behavior of the three studied TEs apparently depends upon many factors, and is controlled at the post-transciptional level. The retrotransposon *gypsyDv* does not transpose, apparently due to accumulation of mutations disturbing *env* or other domains of this TE [31]. However, other authors exploring the PCR technique and PTT analysis concluded that the genome of *D. virilis* may contain at least one copy of *gypsyDv* putatively encoding a complete envelope protein [32] and, hence, we can not exclude that *gypsyDv* may be active in some strains of *D. virilis*. On the other hand, *Ulysses* is transpositionally active in strain 9, probably because in this strain only sense *Ulysses*-piRNAs are present, and the ping-pong cycle is blocked. Along these lines, the ratio of *Ulysses*-derived primary to secondary piRNAs also differs strongly in the strains studied and the low level of *Ulysses* secondary piRNAs in strain 9 may reflect the absence of ping-pong amplification loop necessary for *Ulysses* silencing [22]. Interestingly, in testes a high level of *Ulysses*-derived antisense piRNAs was found, and, surprisingly, this fraction is predominantly homologous to LTRs of this TE (Figure 3A, C). This phenomenon might resemble the different functional activities of Argonaute group proteins in the testes and ovaries [33]. Alternatively, LTR-homologous antisense piRNAs may be coming from a solo *Ulysses* LTRs located in a piRNA-producing cluster functioning only in testes.

Despite the fact, that *Penelope* is one of the most abundant transposon in the genome of *D. virilis* with more than 50 copies in strain 160, we did not detect transpositions of the element to chromosome 6 (microchromosome). This may result from either *Penelope* transposition preferences or from the recently described peculiar chromatin structure of chromosome 6 in *D.virilis* [34], [35]. It is also tempting to speculate that such transposition preferences in avoiding of heterochromatic regions and perhaps piRNA loci might be a reason for a continuing transposition activity of this element in strain 160 of *D. virilis* as well as in transgenic *D. melanogaster* strains transformed with full-size *Penelope* [36].

Comparing the general localization of hybridization sites specific for the studied TEs in the *D. virilis* genome enables us to conclude that the observed distribution is not random, and there are sites where two or three TEs are found. Probably these sites (*e.g.,* 19D and 49F) represent “hot spots” or “nests” of transposons previously described both in the *D. virilis* and *D. melanogaster* genomes [18], [37]. In particular, we do not rule out that at least one of such hot spots, i.e. 49F that coincides with the coordinates of cluster #3 [22], might serve as a putative *flamenco* piRNA locus in *D. virilis* genome that produces the most abundant fraction of sense oriented transposon-homologous piRNAs in *D. virilis* genome.

In the present investigation we did not monitor intrastrain transposition of other TEs mobilized by dysgenic crosses which may represent another interesting avenue of future research, because there are at least two other elements, *Paris* and *Helena,* which are abundant in strain 160, but absent or found in small numbers in strain 9 [12], [17].

Recently, based upon the analysis of maternal inheritance of small RNAs in various systems of *D. melanogaster* HD, it was suggested that piRNAs have an important role in the regulation of the syndrome by homology-dependent TE silencing [25], [27]. In *D. virilis Penelope*-derived small RNAs were also implicated in HD syndrome regulation [22], [28]. Moreover, we speculate that *Penelope* is transpositionally active in strain 160, because, for some reason in this particular quite exceptional strain, small RNAs are represented predominantly by siRNAs. The detected siRNAs probably originated from double stranded stem regions of *Penelope* transcripts containing the same regions in sense and antisense orientation (long inverted repeats). Although siRNAs represent the major class of small RNAs homologous to *Penelope,* it is evident that this class of small RNA is not efficiently transported from mother to embryo and probably does not play any role in *Penelope* silencing in the germ line [22]. Intriguingly, in whole mount experiments we were not able to detect *Penelope* transcription to somatic follicular cells of the ovaries of strain 160, and thereby a subcellular origin of the *Penelope*-siRNAs remains to be investigated.

Collectively, our studies show that two TEs mobilized in dysgenic crosses, namely *Penelope* and *Ulysses*, are drastically different, both in transposition behavior in the parental strains, subcellelar compartmentalization of the transposon transcripts and their processing into small RNAs. It is necessary to mention that we do not rule out the possibility that the causes of occasional transpositions of TEs taking place in the parental strains might be completely different from the causes of much greater mobilization observed in the progeny of dysgenic crosses between these strains.

Although, the investigation of transcription levels and cellular distribution of the transcripts do not provide in all cases a straightforward explanation for the observed interstrain specific transpositions of several transposons, the obtained results should be taken into account in further attempts to explain the molecular mechanisms underlying the behavior of various retroviruses (latent infection) and transposons in laboratory and geographical strains, as well as to shed light on *D. virilis* HD syndrome and the role of co-mobilization of unrelated TEs in this process.

## Materials and Methods

### *D. virilis* strains

*D. virilis* strain 160 and strain 9 were obtained from the Stock Center of the Institute of Developmental Biology, Moscow. Strain 160 represents an old laboratory strain carrying recessive mutations in all autosomes (b, gp, cd, pe, gl) while wild-type strain 9 was collected about thirty years ago in Batumi (Georgia, Caucasus).

All flies were reared at 25°C on standard resin-sugar-yeast-agar medium containing propionic acid and methylparaben as mold inhibitors.

### Cytological analysis

Larvae were grown at 18°C on medium supplemented with live yeast solution for 2 days before dissection. Salivary glands from third instar larvae were dissected in 45% acetic acid and squashed. Procedures and labeling of DNA probes for in situ hybridization were as described [18].

### Whole-mount RNA *in situ* hybridization assay

Ovaries were dissected in PBS and fixed with 4%-paraformaldehyde/PBS solution for 20 min at RT. Treating of ovaries with 20 ug/ml ProteinaseK/PBS solution for 30 min was followed by fixation in 4%-paraformaldehyde/PBS solution for 20 min at RT. During these steps PBS with 0.1% Tween-20 (PBT) was used as a rinsing solution. Pre- and hybridization steps were done at 60°C in HB (50% formamide, 5xSSC, 0.1% Tween 20, 1 mg/ml torula RNA and 50 ug/ml heparin). Antibodies used were anti-DIG-AP (Roche) with 1∶2000 dilution. DIG-labeling of RNA probes was done by MAXIscript T7 kit (Ambion). To detect sense transcripts of studied transposons we used same probes as for Northern blotting (see below), except for *Penelope*:

*pen*623-f: 5′-AGGTCGCCAGAGCCATCAAT-3′;

T7*pen*1264-r: 5′-GCTGATTGGGAGAGCGAACT-3′.

### Northern blotting

Total RNA was isolated from ovaries of 7–10 days old flies using TRIzol reagent (Sigma). PolyA-RNA was purified using OLIGOTEX mRNA mini kit (QIAGENE) and fractionated as described [38]. High Range RNA Ladder (Promega) was used as marker. 32P-labeled single stranded RNA probes revealing sense transcripts were synthesized using MAXIscript T7 kit (Ambion).

Probes for T7 in vitro transcription were synthesized by PCR using:

*gypsyDv*-f: 5′-AGTGGAATTGGCGCGGTTCTTT-3′;

T7*gypsyDv*3983-r: 5′-TAATACGACTCACTATAGGGGCCCATCTTCGAGAGCATTAA-3′;

*uly*5147-f: 5′-CTTCCGCAGACGCAGGATTA-3′;

T7*uly*5698-r: 5′-TAATACGACTCACTATAGGGAGAAATCTGCGCTTCACGCT-3′

### Semiquantitative reverse transcription analysis (RT-PCR) and quantitative real-time PCR (qRT-PCR)

The analyses were performed using 1 ug of DNase I (Fermentas) treated total RNA from ovaries or carcasses. cDNA was prepared using First Strand cDNA Synthesis Kit and random hexamer primers (Fermentas). 2 ul of 5-fold diluted cDNA were used in 30 ul Taq-polymerase PCR mix (SibEnzyme) with 35 amplification cycles.

qRT-PCR was done with 3 biological replicates and carried out using 5x SYBR Green PCR Master Mix (Evrogen) in accordance with the manufacturer's protocol. Quantification was normalized to the endogenous *rp49* and calculation of relative expression levels was done using the 2^-ddCt^ method.

Primers used in the study:

q-uly6798-f: 5′-AAGGAATGCCTAGCCGCCAAA-3′

q-uly6958-r: 5′-AACGCTTGCAGTTCGAGGGA-3′

q-gypsy6113-f: 5′-ACACGTTGGCGGAATGCGAAA-3′

q-gypsy6254-r: 5′-TGAGTGTGGCAGTTGGCGATG-3′

q-paris-f: 5′-ACGGACCCAGCAAAGTTTGGAGAA-3′

q-paris-r: 5′-AGCTCACCAACACCTTTCGACGAT-3′

q-penelope-f: 5′-ACGGTGAGGAGCTAGTGCAAACAA-3′

q-penelope-r: 5′-TTCGTGTCTGTTCCACTGTGTCCA-3′

q-helena-f: 5′-TGGCTCTATGGAGTGCAGATTTGG-3′

q-helena-r: 5′-TCGACTGTGTGCACTTTGAGGTCT-3′

dvir_rp49-f: 5′-TTACGGTTCCAACAAGCGCACC-3′

dvir_rp49-r: 5′-GCGCTCAACAATCTCCTTGCGT-3′

### Small RNA libraries

GEO accession number: GSE22067
